# Autoimmune Hepatitis Triggered by Anti-TNF-**α** Therapy

**DOI:** 10.1155/2013/561748

**Published:** 2013-09-08

**Authors:** Satoshi Nakayama

**Affiliations:** Department of Gastroenterology, Mishuku Hospital, 5-33-12, Kamimeguro, Meguro-ku, Tokyo 153-0051, Japan

## Abstract

Autoimmune hepatitis (AIH) is occasionally triggered by drug treatments. Recently, as biological agents are becoming widely used for autoimmune disorders, there have been a growing number of reports of the development of autoimmune processes related to these agents. A 52-year-old Japanese woman with psoriasis developed liver damage two months after initiation of anti-TNF-**α** therapy with adalimumab. Liver histological findings were compatible with AIH, and positive conversions of ANAs were detected. The patient was treated with prednisolone and had a good response. While some cases of AIH triggered by anti-TNF-**α** therapies have been reported, the pathogenesis remains unspecified. When elevation of liver enzymes is observed with high IgG levels and seropositivity of ANA during the course of anti-TNF-**α** therapy, liver biopsy findings may be essential and important to make definitive diagnosis of AIH.

## 1. Introduction

Approximately 9% of the cases of autoimmune hepatitis (AIH) are triggered by drugs [1]. Recently, as biological agents are becoming widely used for autoimmune disorders, there have been a growing number of reports of the development of autoimmune processes related to biological agents [2]. According to the registry data of the BIOGEAS project (a Spanish registry devoted to collecting data on the use of biological agents in adults with systemic autoimmune diseases), up to 2009, more than 800 cases developed autoimmune diseases associated with biological agents [2]. While drug-induced lupus, vasculitis, optical neuritis, interstitial lung disease, and inflammatory ocular disease accounted for more than 50% of new-onset autoimmune disorders, AIH also accounted for 2.0% [2].

## 2. Case Report

A 52-year-old Japanese woman with a 12-year history of psoriasis started anti-TNF-*α* therapy with adalimumab for exacerbation of skin lesions. The patient was not a habitual drinker and had no concomitant medications at the onset of the therapy. Hepatobiliary enzymes prior to therapy were within normal limits: asparate aminotransferase (AST) 20 U/L (normal value 8–38), alanine aminotransferase (ALT) 24 U/L (normal 4–44), and *γ*-glutamyl transferase (GGT) 43 U/L (normal 16–70). Hepatitis B surface antigen and anti-hepatitis C virus antibody were negative. Antinuclear antibodies (ANAs) measured using ELISA were also negative, 9.7 index (cutoff < 20). 

**Figure 1:**
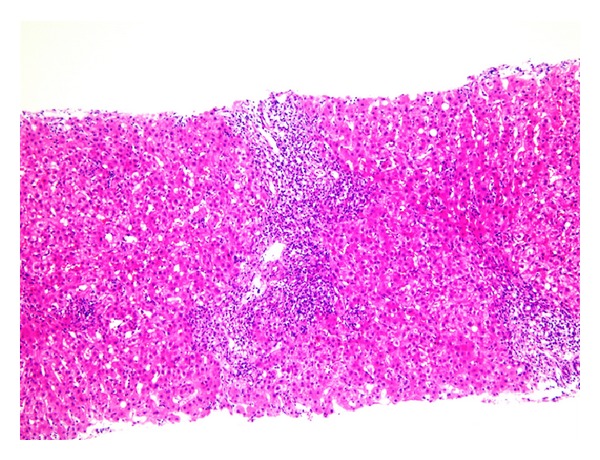
Liver biopsy. Marked portal lymphoplasmacytic inflammation with periportal interface hepatitis and scattered lobular necroinflammatory changes may be seen. (hematoxylin and eosin; ×100).

Two months later, after six doses of adalimumab, she developed malaise with liver damage that continued after the discontinuation of adalimumab. A blood test three months after discontinuation showed the following: AST 240 U/L, ALT 224 U/L, alkaline phosphatase 723 U/L (normal 104–338) and GGT 228 U/L. Whilst serological tests for hepatitis A, B, and C viruses, Epstein-Barr virus, and cytomegalovirus were all negative, her serum immunoglobulin G levels were elevated (1969 mg/dL; normal 870–1700) and homogeneous and speckled-type ANAs were both present at titers of 1 : 160. Anti-smooth muscle antibody, anti-mitochondrial antibody, and anti-DNA antibody were negative. A percutaneous liver biopsy four months after discontinuation of adalimumab showed marked portal lymphoplasmacytic inflammation with periportal interface hepatitis and scattered lobular necroinflammatory changes (Figure 1). Accordingly, we diagnosed the patient as AIH and this was confirmed using simplified international diagnostic criteria (score 8 points; definite AIH ≥ 7) [3]. The patient was treated with 30 mg of prednisolone per day (0.5 mg/kg/day: standard initial therapy regimen for AIH in Japan) for 2 weeks with the reduction of the dose by 5 mg every 2 weeks, and the maintenance dose was set at 5 mg per day. Two months later, her hepatobiliary enzymes returned to normal. While the patient is still under the maintenance therapy, the relapse of AIH has not been observed.

## 3. Discussion

Some 20 cases of AIH triggered by anti-TNF-*α* therapy have been reported to date [3]. We have summarized the seven psoriatic cases with anti-TNF-*α* induced AIH, among which two patients were treated with adalimumab (Table 1) [4–8]. The median time and the number of doses of anti-TNF-*α* drugs to the onset of liver damage were 2 months and 3 times, respectively. All cases discontinued anti-TNF-*α* therapy after the onset of liver damage and six were treated with corticosteroid, with or without azathioprine. All cases had good responses to the therapies and prognosis; the liver damage was resolved within approximately 3 months in five cases. The same tendency was also seen in the cases other than the psoriasis reported previously [3]. 

One report suggested that it is sometimes a challenge to distinguish drug-induced AIH from de novo AIH or drug-induced liver injury because the clinical, biochemical, serological, and histological patterns may be similar in all these groups [9]. Furthermore, patients treated with biological agents may have various forms of simultaneous autoimmune disease and ANAs before treatment [9]. Certain data also suggested that 3% of AIH cases had psoriasis as a concurrent autoimmune disease [10]. 

Our patient could be diagnosed with AIH by positive conversion of ANAs after anti-TNF-*α* therapy and liver histological findings. In particular, liver biopsy findings must have been important to make definitive diagnosis of AIH. 

The pathogenesis of AIH triggered by anti-TNF-*α* therapy remains unspecified [4]. TNF-*α* itself is suspected of contributing to the development of AIH [11], and an AIH case treated with anti-TNF-*α* inhibitor has also been reported [12]. In a certain case, AIH did not relapse after switching to another anti-TNF-*α* drug [13]. Perhaps, like other drugs, reactive metabolites of the anti-TNF-*α* drugs, which can be recognized by the immune system as neoantigens, may be one of the causes of AIH [1]. 

Occasionally, some patients may develop AIH after starting anti-TNF-*α* therapy for autoimmune disorders. Perhaps, most of them will develop AIH few months after initiation of the treatment and this will be resolved by intensive immunosuppressive therapy. Therefore, routine liver function test may be recommended during the course of anti-TNF-*α* therapy. When elevation of liver enzymes are observed with high IgG levels and seropotivity of ANA, liver biopsy findings may be essential and important to make definitive diagnosis of AIH.

## Figures and Tables

**Table 1:** Reported cases of psoriasis with AIH triggered by anti-TNF-*α* therapy.

No.	Sex	Age (years)	Anti-TNF-*α*	Time to liver damage Number of doses	Expressed autoantibodies	Treatment	Time to the resolution of liver damage
1 [4]	Female	53	Infliximab	26 weeks 6	ANA Anti-ds DNA ASMA	Fluocortolone	12 weeks
2 [5]	Female	22	Infliximab	6 weeks 2	ANA	Prednisone Azathioprine	NA
3 [6]	Male	37	Infliximab	6 weeks 3	ANA Anti-ds DNA ASMA	Cessation only	2 months
4 [7]	Male	51	Infliximab	One month 3	ANA Anti-ds DNA AMA Anti-cardiolipin antibodies	Azathioprine Corticosteroids Ursodeoxycholic acid	NA
5 [8]	Female	36	Adalimumab	3 months 6	ANA Anti-ds DNA	Prednisone Azathioprine	2 months
6 [8]	Female	47	Infliximab	10 weeks 3	ANA ASMA	Prednisolone	3 months
7 (our case)	Female	52	Adalimumab	2 months 6	ANA	Prednisolone	2 months

ANA: anti-nuclear antibody, Anti-ds DNA: anti-double stranded DNA antibody, ASMA: anti-smooth muscle antibody, AMA: anti-mitochondrial antibody, PBC: primary biliary cirrhosis, and NA: not available.
